# Axially evoked postural reflexes: influence of task

**DOI:** 10.1007/s00221-014-4105-8

**Published:** 2014-10-10

**Authors:** Sendhil Govender, Danielle L. Dennis, James G. Colebatch

**Affiliations:** 1 Prince of Wales Clinical School and Neuroscience Research Australia, University of New South Wales, Randwick, Sydney, NSW 2031 Australia; 2 Institute of Neurological Sciences, Prince of Wales Hospital, Randwick, Sydney, NSW 2031 Australia

**Keywords:** Postural reflexes, Axial reflexes, Spinal reflexes

## Abstract

Postural reflexes were recorded in healthy subjects (*n* = 17) using brief axial accelerations and tap stimuli applied at the vertebra prominens (C7) and manubrium sterni. Short latency (SL) responses were recorded from the soleus, hamstrings and tibialis anterior muscles and expressed as a percentage of the background EMG prior to stimulus onset. In the majority of postural conditions tested, subjects were recorded standing erect and leaning forward with their feet together. The SL response was larger for soleus than for the hamstrings during standing (soleus vs hamstrings; 70.4 vs 28.1 %), whereas the opposite occurred during kneeling (25.3 vs 127.3 %). Concordant head and trunk accelerations produced larger SL responses than discordant accelerations for soleus and hamstrings, but the evoked excitatory response was independent of head direction and as expected for the direction of truncal acceleration. Postural reflexes for soleus and tibialis anterior were strongly affected by conditions that posed a significant threat to postural stability; stimulation at C7 was associated with significant SL enhancement for soleus during anterior lean while sternal stimulation showed SL enhancement for tibialis anterior during posterior lean. Cutaneous anaesthesia applied over the C7 stimulation site had no significant effect on EMG responses, nor did vision or surface type (rigid or compliant). This study provides further evidence that postural reflexes produced by brief axial accelerations are independent of cutaneous receptors, vestibular afferents and ankle proprioceptors, and demonstrates that postural tasks and truncal orientation significantly affect the evoked response, consistent with a role in stabilising posture.

**Electronic supplementary material:**

The online version of this article (doi:10.1007/s00221-014-4105-8) contains supplementary material, which is available to authorized users.

## Introduction

Unexpected perturbations when standing present a threat to postural stability and short latency (SL) reflexes have an important role in countering such threats. Short latency reflexes in leg and trunk muscles have been demonstrated to arise from vestibular receptors in response to head accelerations (Horak et al. 1994; Laube et al. 2012) and following electrical vestibular stimulation (Britton et al. 1993; Fitzpatrick et al. 1994). Sudden surface displacements, similar to a slip, also evoke SL postural reflexes, which are prominent in trunk muscles (Horak et al. 1994). While these responses have been attributed to afferents excited by ankle movements (Fitzpatrick et al. 1994), postural reflexes arising from truncal receptors have also been reported (Gurfinkel et al. 1981; Bloem et al. 2000, 2002) but not widely known or accepted. Recently, Graus et al. (2013) presented further evidence in support of an axial source of postural reflexes. These authors applied small perturbations to the head and trunk and showed that the responses in soleus were determined by the direction of the applied disturbance, that the upper trunk was the most effective site of stimulation and that the responses were not present with the subjects seated. Graus et al. (2013) also reported that a brief acceleration to the upper trunk evoked postural reflexes in the soleus muscles that inverted when the direction of trunk acceleration was changed, confirming a previous report by Bőtzel et al. (2001). Graus et al. (2013) argued that, even though a similar acceleration applied to the mastoids evoked vestibular-dependent reflexes in the legs with similar latencies, the response to truncal accelerations was not primarily mediated through vestibular receptors. Their arguments included the limited changes in the reflexes seen in vestibular patients. Like previous reports (Do et al. 1988; Allum et al. 1995; Bloem et al. 2000, 2002), it was thought that the responses could not be primarily arising from lower leg proprioceptors, but more likely from truncal or hip proprioceptors, consistent with the optimum effects seen with upper truncal stimuli.

The present experiments were designed to provide further evidence as to the origins and properties of these reflexes. One specific object was to dissociate the direction of head and trunk acceleration. Normally, these are highly correlated (Graus et al. 2013) making it difficult to assess the contribution from vestibular receptors. We also wished to investigate the nature of the reflex in other leg muscles, as well as the response to differing postural tasks.

## Methods

### Study participants

Seventeen healthy subjects (mean age 25 ± 10 years, ten males and seven females) with no history of inner ear pathology were recruited from the general population and from staff at the Prince of Wales Hospital, Sydney. Subjects gave written consent according to the Declaration of Helsinki, and the study was approved by the local ethics committee.

### Stimulation techniques

Subjects were stimulated with impulsive acceleration and taps applied over the spine of the C7 vertebra (“C7”—see Graus et al. 2013), or the sternum. The smoothed impulsive stimulus consisted of a third-order gamma distribution (Ross 2007) with a 12-ms rise time. The stimulus was delivered using a customised minishaker (model 4810, Brüel and Kjaer P/L, Denmark) with an attached perspex rod (diameter 2.5 cm, length 9.2 cm). A second minishaker, used in the dual-stimulus conditions, was mounted on a free-standing frame with a 1-kg counterweight. This minishaker was mounted on an arm and was free to rotate in the horizontal plane with the counterweight ensuring that a consistent force was applied to the subjects’ foreheads. The waveform was generated using customised software through either a micro1401 or a 1401plus laboratory interface (CED, Cambridge UK) and amplified. The movement of the perspex rod was either in the ‘positive’ or ‘negative’ direction, defined as movement of the rod away from or towards the motor, respectively. Impulsive stimuli were delivered at a driving voltage of 10 V peak (0 dB; equivalent to approximately 6.6 N peak force) and 20 V peak (+6 dB; equivalent to approximately 10 N peak force). Stimuli were delivered at a rate of ~2.5 Hz. Taps were delivered using an electronically triggered reflex tendon hammer (Nicolet Biomedical, WI, USA).

### Recordings

Self adhesive electrodes (Cleartrace 1700-030, Conmed Corp., NY, USA) were used to record rectified and unrectified EMG from over the soleus muscles bilaterally and from either the hamstrings or tibialis anterior muscles. For the soleus muscles, recorded under all conditions, reference electrodes were positioned on the posterior aspect of the lower leg over the Achilles tendon while the active electrodes were positioned approximately 6 cm above. For the hamstrings, recorded in all but the three leaning conditions, the active electrodes were positioned medial to the midpoint of the posterior thigh muscles (between the ischial tuberosity and the popliteal fossa) while the reference electrodes were positioned approximately 6 cm below this. For the TA muscles, recorded during differing leaning postures, the active electrodes were placed lateral to the upper third of the tibia with the reference electrodes approximately 6 cm below.

An earth electrode was positioned on the right or left forearm, 5 cm distal to the antecubital fossa. Recordings were made from 50 ms prior to stimulus onset to 250 ms afterwards. EMG was amplified, bandpass filtered (8–1,600 Hz) and sampled using a CED Power1401 laboratory interface and recorded using Signal software (version 3.13, Cambridge Electronic Design, Cambridge, UK). Both unrectified and rectified averages were made, but quantitative measurements were made in most cases using the averaged, full-wave rectified EMG.

Evoked sway was measured using a force platform (model 9286A, Kistler Instrumente, Winterthur, Switzerland). Data were sampled at 4 kHz and collected using a CED Power1401 laboratory interface and recorded using Signal software. Centre of pressure (CoP) was calculated for the anteroposterior (AP) plane using the force platform manufacturer’s formula and custom Matlab software (Mathworks, MA, USA) and averaged.

### Experimental protocols

#### Effects of cutaneous anaesthesia of the site of stimulation

This was studied for eight subjects (mean age 29 ± 12 years, five males and three females), with several participating in other sets of observations (*n* = 6). The experimental protocol consisted of positive impulses (at two intensities: 0 and +6 dB) and tendon hammer taps applied over C7 while both standing and kneeling. After completion of the baseline observations, local anaesthetic was applied over C7 either topically (*n* = 5) or via subcutaneous injection (*n* = 3). Topical anaesthesia was applied over a 6 × 7 cm area using 5 % EMLA cream (AstraZeneca Australia, North Ryde) and secured with Tegaderm transparent film dressing (3 M Health Care, MN). Subcutaneous administration was carried out using 3–4 ml of 2 % lignocaine hydrochloride (Pfizer Australia, West Ryde) injected to 4–6 sites around the usual stimulation site. Skin sensation was assessed approximately 45–60 min postapplication using neurological examination pins (Neurotips, Owen Mumford Inc., GA). Overall, the anesthetised region was approximately 5 cm × 6 cm and included the area of the applied stimuli. The subjects were then retested.

#### Dissociation of head and trunk accelerations

Experiments were performed in ten subjects (mean age 24 ± 5 years, six males and four females). For the standing conditions, subjects stood with their feet comfortably apart and were asked to lean forward to activate their soleus muscles and, except where specified, to close their eyes. For all the conditions, the primary minishaker was hand-held and applied to the trunk over C7. To dissociate head from trunk acceleration, four dual-stimulus standing conditions were studied in which a positive impulse was applied over C7 while a second impulse was applied simultaneously to the centre of the subject’s forehead, this impulse being generated using the second minishaker mounted on the rotating arm of the frame. This stimulus, applied to the forehead, was kept at a constant intensity (0 dB) but for two of the conditions the initial direction was positive and for two it was the reverse. The stimulus at C7 always delivered a positive impulse but two intensities were used, giving a total of four stimulus combinations (two concordant and two discordant directions of acceleration).

#### Effects of vision, kneeling and surface compliance

For this part of the study, impulsive stimuli were applied with a positive initial acceleration over C7 at two intensities (0 dB eyes closed; +6 dB eyes closed and open) and taps were applied to the same location at a rate of approximately 2 Hz. For the compliant surface, subjects stood on a 12-cm-thick latex foam cushion and had three different stimuli applied over C7 (two impulse intensities and taps). The effect of kneeling was investigated using impulsive (0 and +6 dB) and tendon hammer stimuli applied at C7. For this condition, subjects knelt on a stool at a height of 45 cm and closed their eyes. They were prompted to activate their soleus muscles by plantar flexing their feet.

#### Effects of differing amounts of postural lean

A final session examined EMG responses in the soleus and TA muscles to the impulsive stimulus and tendon hammer taps for eight subjects (mean age 28 ± 13 years, four males and four females), two of whom participated in both other sessions while one subject also participated in the cutaneous anaesthesia experiment. Subjects held one of three postures—anterior lean, neutral stance, or posterior lean. Subjects were asked to either stand naturally or lean as far as possible forwards or backwards, keeping a straight back, without lifting any part of their foot off the ground and with their eyes closed. The positive impulses (at two intensities: 0 and +6 dB) and tendon hammer taps were applied over C7 for each posture, and the same was repeated with impulse stimulation over the sternum. Taps were not applied to the sternum as this was previously found to be poorly tolerated (see Graus et al. 2013). A sway surrogate (strictly, CoP) was also measured for this experimental component using the force platform. Subjects stood on the platform and foot outlines were marked on the platform as a guide for subjects’ foot placement. To determine each subject’s approximate maximum level of tonic activation, brief recordings (~50 trials; eyes open, no stimulus) were made while subjects contracted their soleus and TA muscles bilaterally by standing on their toes and heels, respectively.

All impulsive conditions consisted of 120–200 individual trials, and all tap conditions of 80 individual trials from which subject averages were made. The order of conditions presented was randomised between subjects.

### Accelerometry

Head and trunk acceleration were recorded during the experiment using two uniaxial accelerometers (Endevco 751-100). One accelerometer was positioned over the sternum or C7 while the other was positioned over the occiput. The dominant initial peak was used for amplitude and latency measurements.

### Analysis

Amplitude and latency values were analysed from rectified averaged EMG traces using customised software. Response amplitudes, which scale in proportion to background activation (Bőtzel et al. 2006), were calculated as the change in the mean rectified EMG and expressed as a percentage of the background EMG activation prior to stimulus onset (Graus et al. 2013; Welgampola and Colebatch 2001). Latency values were taken at the onset of the initial excitation (SL), the end of the SL excitation [start of the medium latency (ML) inhibition] and end of the ML inhibition. As the inhibition may be simply recovery from the preceding excitation (Graus et al. 2013), we have focused primarily on the SL excitability changes.

Statistical analysis was conducted using SPSS (version 22.0, IBM Inc., Chicago, USA). There was no significant difference at baseline between right and left mean rectified EMG levels for the soleus or hamstring muscles (*P* > 0.05). Therefore, results for the two sides have been combined for analysis. For amplitudes, ANOVAs were performed using a within-subject design for each muscle group (soleus and hamstrings). For absent or missing values for amplitudes or latencies, a between-subjects design was used. Paired *t* tests were used to compare the effect of vision. Surface type (rigid and compliant) and stimulus type (0 dB, +6 dB and taps) were used as factors when analysing the effect of surface compliance. For kneeling, ANOVAs were performed for response amplitudes and, when comparing the effect of kneeling to standing, the two factors were posture (kneeling and standing) and stimulus type. For dual stimulation, the direction of head acceleration (anterior and posterior) and intensity (0 and +6 dB) were used as factors. Similar methods were also used for comparing acceleration amplitudes and latencies. The responses of one subject during cutaneous anaesthesia in the kneeling condition were inverted compared to the other subjects, most likely due to a difference in how the C7 stimulus was applied causing him not to lean forwards as far as other subjects did. This subject’s data were excluded from all kneeling analyses. The effect of cutaneous anaesthesia was analysed using treatment (pre and post) and stimulus type (impulsive 0 dB, impulsive +6 dB and taps) as the two factors. Responses during different degrees of voluntary lean for a given location of stimulation were analysed using muscle group (soleus and tibialis anterior) and leaning posture (anterior, neutral and posterior) as factors. Post hoc *t* tests were used to compare amplitude and latency values when significant main effects or interactions were found on ANOVA. For multiple comparisons, the level of significance (*P* < 0.05) was adjusted using the Bonferroni correction. We have noted in the text when *p* values fell below 0.05 but failed to reach the Bonferroni corrected *p* value. Positive and negative percentages indicate excitation and inhibition, respectively. Values in the text and tables are given as mean ± SD.

## Results

### Baseline properties

**Fig. 1:**
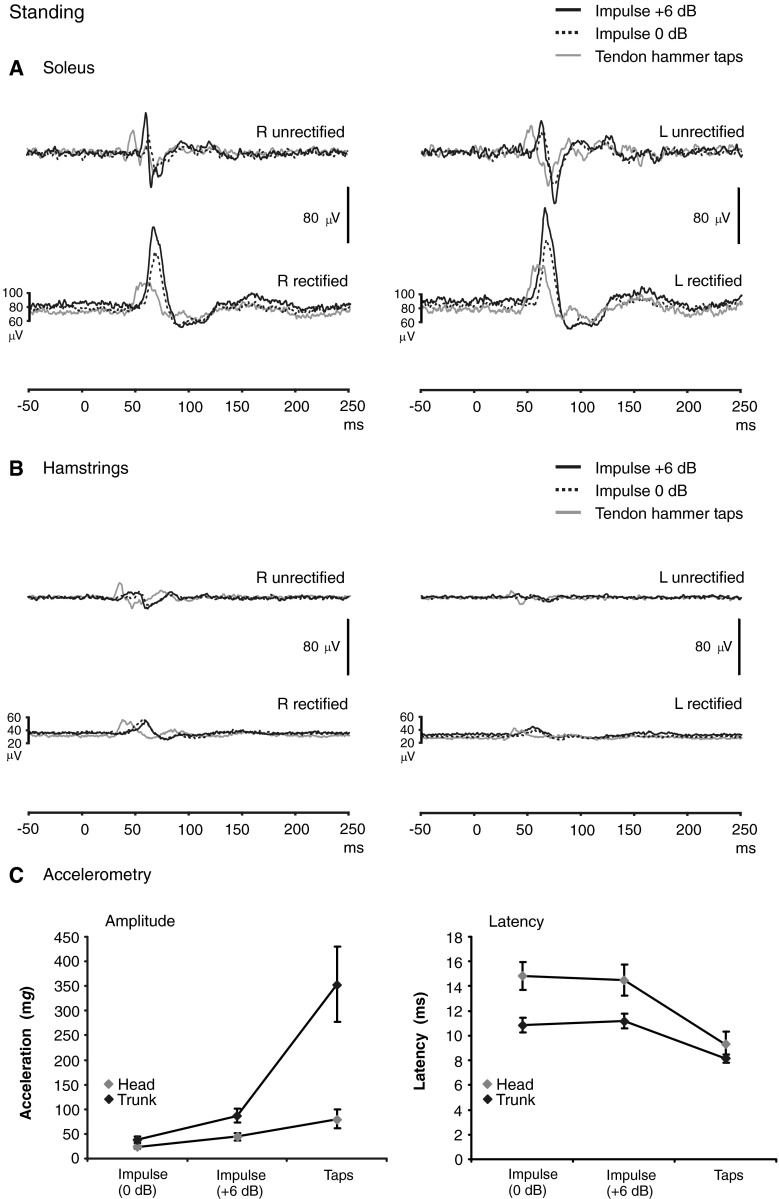
Baseline conditions—grand average EMG traces, soleus (**a**) and hamstrings (**b**), showing responses to all three modalities of stimulation. In all cases, the subjects were standing, eyes closed, leaning forwards slightly to tense the soleus muscles. Unrectified averages are shown above the rectified ones. In most subjects, a biphasic response was present on the unrectified averages. Due to latency differences between subjects, the peak-to-peak amplitude of the grand mean biphasic response (illustrated: 131 μV) was less than for the mean of the individual values (329 μV). Quantitation was done using the rectified averages, which showed an excitation followed by an inhibition. The impulsive stimuli gave larger responses in soleus despite the tendon hammer tap inducing a higher acceleration at the sternum than the minishaker stimulus (**c**). Standard errors are shown in part **c**

As previously reported (Graus et al. 2013), the tendon hammer taps evoked the largest accelerations, especially for the trunk, and the +6 dB impulse evoked a larger acceleration than the 0 dB one (Supplementary Table: *P* < 0.001). The responses in soleus, with the subjects standing with eyes closed and leaning forwards on a rigid surface, were similar to the report of Graus et al. (2013). For a positive (anterior) acceleration, the response in soleus consisted of an initial SL excitation followed by an ML inhibition evident on the average rectified traces and corresponding to a biphasic volley often evident (present in 9/10 subjects) on the unrectified average (Fig. 1: mean peak-to-peak amplitude of 329 μV).

For our subjects standing, we observed similar responses in soleus to those previously reported, an average initial SL excitation of 70.4 % for the 0 dB impulse, 92.8 % for the +6 dB impulse, and 55.4 % for the tendon hammer. Mean rectified values for soleus varied between 72.3 and 87.5 μV.

The hamstring responses showed similar but lower amplitude excitability changes to those in soleus, with a biphasic volley sometimes evident on the unrectified averages and an initial excitation and inhibition on the average rectified EMG. Mean rectified values for the hamstrings (0 dB: 33.0 ± 17.6 μV, +6 dB: 35.1 ± 15.6 μV, taps: 27.0 ± 11.2 μV) were significantly smaller than for soleus (*P* ≤ 0.002). Averaged SL amplitudes evoked by the impulsive stimuli when standing on the rigid surface, even allowing for the lower level of tonic activation, were significantly smaller than for soleus (*P* ≤ 0.002). Corrected amplitudes in the hamstrings were, however, larger following the tendon hammer tap and similar to those in soleus for the same stimulus (Table 1). Average latencies of the reflexes from the hamstrings were shorter than for soleus for all three measurements (*P* ≪ 0.001 with a mean difference of 11.3 ms for the SL excitation onset during standing; Table 2).

**Table 1:** EMG response amplitudes (%) for postural reflexes in the standing and kneeling conditions

	SL excitation				ML inhibition			
	Impulsive stimulus (0 dB)	Impulsive stimulus (+6 dB)		Taps	Impulsive stimulus (0 dB)	Impulsive stimulus (+6 dB)		Taps
	EC	EC	EO	EC	EC	EC	EO	EC
*Soleus EMG*								
Standing (rigid surface)	70.4 (38.3)	92.8 (43.0)	85.3 (33.5)	55.4 (24.4)	−26.0 (5.8)	−30.9 (7.4)	−27.3 (6.6)	−26.2 (11.0)
Standing (compliant surface)	79.2 (54.4)	100.6 (51.9)	–	72.8 (35.9)	−27.8 (8.6)	−29.8 (8.3)	–	−29.2 (26.6)
Kneeling	25.3 (32.7)	40.6 (50.2)	–	25.7 (22.4)	−11.5 (5.5)	−15.7 (5.5)	–	−7.6 (8.1)
*Hamstrings EMG*								
Standing (rigid surface)	28.1 (19.4)	31.3 (13.8)	30.3 (13.3)	55.8 (29.0)	−14.4 (10.6)	−16.7 (11.1)	−15.6 (12.5)	−22.3 (15.7)
Standing (compliant surface)	25.7 (12.1)	34.1 (17.2)	–	67.6 (47.2)	−16.0 (8.7)	−18.8 (7.2)	–	−20.0 (12.6)
Kneeling	127.3 (75.4)	146.7 (74.8)	–	99.9 (68.1)	−29.3 (25.4)	−36.6 (13.2)	–	−9.0 (14.4)

*EC* eyes closed; *EO* eyes open; all amplitudes are expressed as a percentage change compared to prestimulus EMG levels, and values are expressed as mean (SD)

**Table 2:** EMG response latencies in the standing and kneeling conditions

	Impulsive stimulus (0 dB)			Impulsive stimulus (+6 dB)						Taps		
	EC			EC			EO			EC		
	Excit onset	Inhib onset	Inhib end	Excit onset	Inhib onset	Inhib end	Excit onset	Inhib onset	Inhib end	Excit onset	Inhib onset	Inhib end
*Soleus EMG*												
Standing (rigid surface)	58.3 (2.8)	77.6 (3.0)	117.7 (8.4)	56.6 (3.4)	77.8 (3.2)	122.4 (5.9)	57.0 (3.2)	77.2 (4.1)	121.0 (7.8)	50.6 (2.9)	74.7 (6.3)	100.6 (20.5)
Standing (compliant surface)	58.7 (3.7)	80.1 (2.4)	118.4 (12.6)	57.3 (3.2)	79.5 (3.8)	127.2 (12.4)	–	–	–	51.3 (4.6)	78.7 (4.1)	106.6 (16.2)
Kneeling	57.9 (4.0)	76.2 (4.5)	96.1 (10.2)	57.5 (3.6)	75.4 (5.2)	100.1 (18.3)	–	–	–	53.0 (5.0)	71.9 (4.0)	86.8 (8.4)
*Hamstrings EMG*												
Standing (rigid surface)	47.0 (3.7)	68.8 (3.1)	95.2 (11.6)	44.8 (4.4)	66.5 (3.0)	96.3 (19.6)	41.1 (5.3)	64.6 (4.0)	87.0 (14.9)	36.3 (2.3)	57.8 (5.7)	74.3 (10.1)
Standing (compliant surface)	46.5 (4.5)	68.6 (3.8)	97.6 (13.8)	44.2 (3.4)	67.2 (2.9)	107.1 (21.9)	–	–	–	36.4 (2.7)	58.7 (7.3)	83.2 (20.3)
Kneeling	48.8 (2.1)	72.9 (3.6)	112.1 (7.1)	47.5 (3.0)	72.9 (3.9)	117.7 (13.1)	–	–	–	44.6 (3.1)	71.3 (6.4)	90.9 (17.9)

*EC* eyes closed; *EO* eyes open; latencies are given in ms following the onset of drive to the truncal minishaker or the trigger for the tendon hammer. All values are expressed as mean (SD)

There was no significant difference between pre- and postanaesthesia for either amplitude or latency of postural reflexes (*P* > 0.05, Supplementary Figure). Mean rectified EMG values during standing were also not significantly different (*P* > 0.05).

### Concordant and discordant head and trunk accelerations

**Fig. 2:**
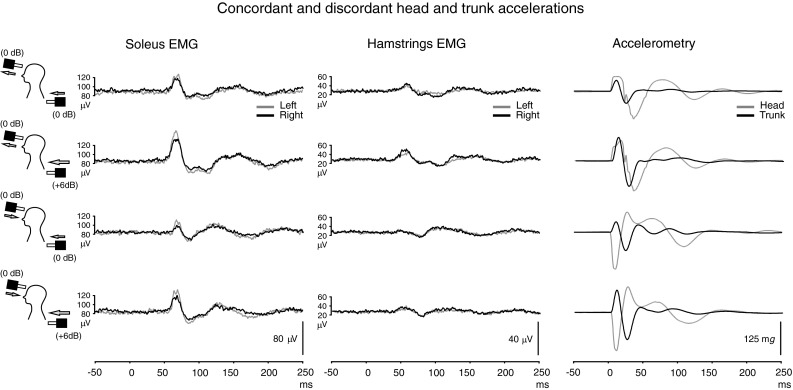
EMG recordings and from the soleus muscles and hamstrings during dual stimulation at C7 and at the forehead (*n* = 10). For accelerometry traces, upward and downward deflections reflect accelerations in the anterior and posterior directions, respectively. Truncal accelerations were always in the anterior direction using either a 0 or +6 dB stimulus intensity applied at C7. Head accelerations were either anterior (*top* two traces; same direction as the trunk) or posterior (*lower* two traces; the opposite direction to the trunk) and achieved by changing the stimulus polarity of the minishaker applied at the forehead. Responses were larger with concordant than discordant accelerations. Note that the gain for the hamstrings EMG is twice that of the soleus

We deliberately dissociated the direction of acceleration of the trunk, which was always anterior, from that of the head, which we made either anterior or posterior (Fig. 2; Table 3). In most instances, head accelerations in both directions were greater than that for the trunk [concordant: head (trunk); 0 dB: 63.9 ± 18.4 mg (46.1 ± 23.3 mg); +6 dB: 81.1 ± 32.3 (101.1 ± 49.9 mg); discordant: 0 dB: −154.4 ± 27.9 mg (43.6 ± 22.1 mg); +6 dB: −157.4 ± 33.1 mg (92.9 ± 48.4 mg)]. In all cases, the SL excitation was as expected for the direction of trunk acceleration and not what would be predicted if the direction of the acceleration of the head was the critical factor. For both muscle groups, the average SL responses were larger for the concordant than for the discordant accelerations (*P* ≤ 0.011: Table 3).

**Table 3:** EMG amplitude and latency during concordant and discordant head and trunk accelerations

	Amplitude (%)		Latency (ms)		
	SL (excitatory) response	ML (inhibitory) response	Excit onset	Inhib onset	Inhib end
*Soleus EMG*					
Concordant					
0 dB	35.7 (26.0)	−16.9 (5.1)	57.8 (4.1)	77.8 (3.5)	118.0 (16.6)
+6 dB	59.5 (36.9)	−24.9 (6.2)	60.8 (3.8)	77.5 (2.9)	111.7 (15.9)
Discordant					
0 dB	19.1 (33.7)	−19.5 (6.6)	59.9 (4.9)	77.2 (3.7)	99.3 (6.3)
+6 dB	37.8 (35.6)	−24.4 (7.5)	58.9 (4.6)	77.4 (3.8)	106.9 (9.2)
*Hamstrings EMG*					
Concordant					
0 dB	19.0 (13.3)	−13.5 (7.6)	46.4 (5.9)	71.3 (6.1)	102.2 (16.8)
+6 dB	27.0 (16.8)	−15.4 (8.7)	50.7 (4.6)	71.6 (5.6)	101.2 (17.0)
Discordant					
0 dB	6.3 (5.4)	−16.3 (4.9)	46.0 (6.7)	64.8 (6.9)	84.8 (4.8)
+6 dB	17.4 (10.1)	−16.1 (7.7)	46.8 (6.1)	66.8 (6.7)	84.8 (6.9)

Values are given as mean and (SD), expressed as a percentage change from the preceding tonic level

Concordant = Trunk anterior, head anterior; discordant = trunk anterior, head posterior

There was no significant difference in mean rectified EMG levels for either muscle group between conditions (soleus mean 79.4 ± 38.1 μV, hamstrings mean 30.7 ± 15.5 μV; all *P* > 0.05).

### Vision and surface effects

**Fig. 3:**
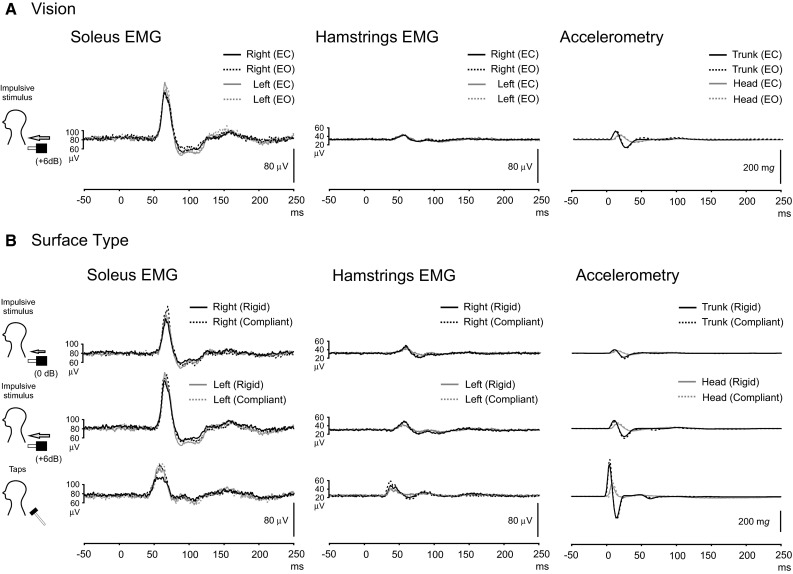
Grand mean recordings showing the limited effects of vision (**a**) and surface compliance (**b**) on soleus and hamstring EMG responses to truncal perturbations (*n* = 10). *EC* Eyes closed, *EO* eyes open. In all conditions, subjects were standing and leant foreword while stimuli were delivered to C7

The effect of vision was assessed using the + 6 dB impulsive stimulus (Fig. 3a). Acceleration amplitudes did not differ between the eyes-closed and eyes-open conditions, whereas acceleration latencies at the head peaked slightly earlier during eye closure (Supplementary Table: *P* = 0.003). Vision had no significant effect on EMG responses from either muscle group for the SL excitation. Mean rectified values for the soleus and hamstrings during the eyes-open condition were 95.4 ± 44.1 and 39.0 ± 18.7 μV, respectively, with no significant effect of vision on rectified EMG levels for either muscle group (*P* > 0.05).

Subjects were tested while standing on a rigid or compliant surface (Fig. 3b). For both muscle groups, the SL excitations were not significantly different using the compliant surface for either muscle group (*P* > 0.05). Mean rectified EMG values were not significantly different between surfaces for either the soleus or the hamstring muscles (*P* > 0.05).

### Responses during kneeling

**Fig. 4:**
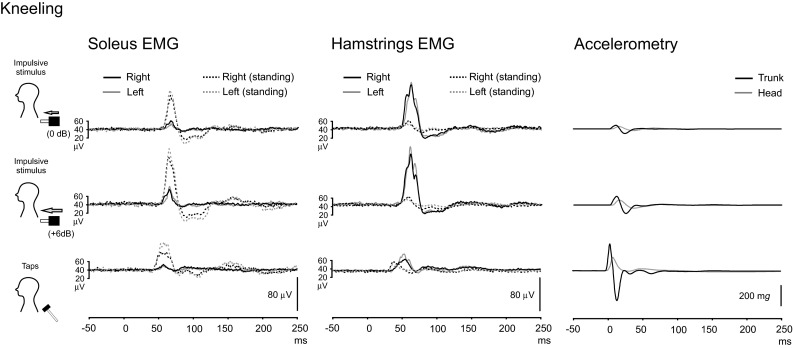
Effect of kneeling on responses from the soleus and hamstring muscles groups (*n* = 10). In contrast to the standing condition (*dashed lines*), kneeling produced much larger responses in the hamstrings compared to the soleus muscles. While soleus responses were smaller when kneeling, they were not abolished. *Baselines* reflect rectified EMG levels for kneeling responses only

When kneeling, accelerations of the head and trunk were of similar amplitudes to those when standing (*P* > 0.05), and again the tendon hammer taps induced greater accelerations for the trunk than the head (Supplementary Table). Hamstring responses for the SL excitation when kneeling was much larger than when standing for all the stimuli (Fig. 4; Table 1). Responses in the soleus muscles, which we had asked subjects to deliberately contract, were present but attenuated by more than half compared to standing. There were significant main effects of posture type on reflex amplitude in the SL excitation in the hamstrings (increased *P* < 0.001) and the soleus muscles (reduced *P* < 0.001). The SL excitation in the hamstrings was significantly larger when kneeling for both impulse intensities (*P* < 0.001). For the hamstrings, there was a main effect of posture for all latencies with slightly longer latencies when kneeling, despite the larger responses (Table 2; *P* < 0.001). An additional feature, clearer for the hamstrings and individual subjects, was that the initial response could sometimes be followed by a second volley of excitation, typically at an average interval of approximately 16 ms. These second volleys were commonly seen for the more difficult postural tasks (kneeling) and with the tendon hammer taps.

Mean rectified levels for this condition were significantly lower for soleus than in the standing condition (soleus kneeling, average for the three stimuli: 39.7 ± 18.3 μV; *P* ≤ 0.011). For the hamstrings, mean rectified levels were generally larger during kneeling but overall not significantly different from the standing condition (hamstrings kneeling, average for the three stimuli: 41.7 ± 26.1 μV; *P* > 0.05).

### Responses during anterior and posterior lean

Overall, CoP average displacement from the neutral position was 15.0 mm for voluntary anterior lean and 20.5 mm for posterior lean. For both C7 and sternal stimulation, acceleration amplitudes were larger for the trunk than the head (*P* < 0.02) and greater for the +6 dB impulse (*P* = 0.001). Truncal acceleration amplitudes were larger for C7 stimulation than sternal during anterior (83.9 ± 58.0 vs 58.3 ± 41 mg), neutral (100 ± 71.4 vs 52 ± 37.2 mg) and posterior lean (110.2 ± 82.2 vs 68.4 ± 59.7 mg; all *P* ≤ 0.002).

**Fig. 5:**
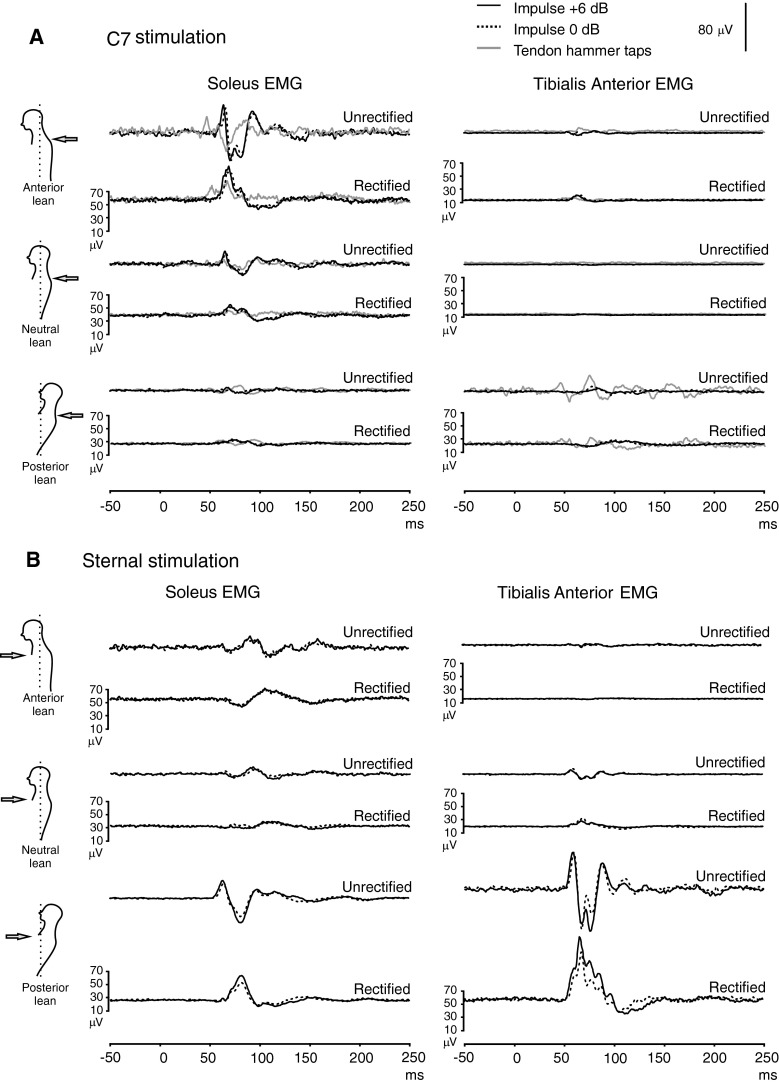
Grand means of postural responses from the soleus and tibialis anterior muscle groups for C7 (**a**) and sternal (**b**) stimulation during anterior, neutral and posterior leaning posture (*n* = 8). Standard (0 dB) and high (+6)-intensity impulsive stimuli were used for both C7 and sternal stimulation, whereas taps were used to elicit responses for C7 stimulation only. Responses in soleus were facilitated during anterior lean and in tibialis anterior, by leaning posteriorly, by a greater degree than explained by the change in tonic activation. For this illustration, both rectified and unrectified EMG traces are shown for the *right side* only. *Left-sided* responses were very similar

Soleus mean rectified EMG was largest with leaning anteriorly and smallest leaning posteriorly while tibialis anterior levels showed the opposite pattern (Table 4). Positive impulsive stimulation at C7 and taps produced an initial SL excitation followed by an ML inhibition in the tibialis anterior for neutral stance and anterior lean, while the responses were very attenuated with posterior lean (Fig. 5a). The largest postural responses in soleus occurred during anterior lean, often demonstrating a biphasic volley on the unrectified trace (present in 7/8 subjects; mean peak-to-peak amplitude of 119 μV). The SL responses demonstrated a significant interaction between muscle group and posture for both impulsive stimuli (0 dB: *P* = 0.002, +6 dB: *P* = 0.009).

**Table 4:** Amplitudes (%) and latencies (ms) for the SL phase and mean background activation levels during leaning

	Soleus EMG			Tibialis Anterior EMG		
	Impulsive stimulus (0 dB)	Impulsive stimulus (+6 dB)	Background activation (μV)	Impulsive stimulus (0 dB)	Impulsive stimulus (+6 dB)	Background activation (μV)
*C7 stimulation*						
Anterior lean	33.7 (16.0)	43.3 (20.0)	**66.1** (**15.5**)	26.8 (15.1)	28.9 (12.7)	**16.7** (**1.6**)
SL onset	*59.4* (*2.1*)	*58.0* (*2.7*)		*58.3* (*3.6*)	*55.9* (*3.2*)	
SL end	*84.9* (*6.9*)	*82.1* (*4.4*)		*76.6* (*6.1*)	*75.2* (*6.4*)	
Neutral lean	20.1 (8.9)	25.8 (15.9)	**41.4** (**14.3**)	9.4 (8.0)	11.8 (9.8)	**14.3** (**1.0**)
SL onset	*61.9* (*4.0*)	*59.4* (*4.3*)		*62.6* (*4.3*)	*61.8* (*5.0*)	
SL end	*87.3* (*5.6*)	*86.0* (*8.1*)		*82.5* (*8.3*)	*85.4* (*9.1*)	
Posterior lean	15.5 (14.0)	20.2 (16.8)	**29.9** (**10.2**)	−9.0 (9.2)	−10.2 (10.5)	**26.0** (**12.8**)
SL onset	*57.6* (*3.3*)	*57.2* (*3.0*)		*57.5* (*4.6*)	*59.0* (*6.0*)	
SL end	*85.1* (*8.9*)	*86.8* (*8.6*)		*82.0* (*8.3*)	*87.1* (*6.1*)	
*Sternal stimulation*						
Anterior lean	−11.5 (10.7)	−14.3 (10.0)	**54.6** (**17.7**)	−0.1 (3.9)	0.7 (3.4)	**14.5** (**0.8**)
SL onset	*65.6* (*5.3*)	*63.5* (*3.6*)		*61.2* (*0.4*)	*61.9* (*1.3*)	
SL end	*91.7* (*4.7*)	*93.6* (*3.4*)		*90.5* (*2.6*)	*95.1* (*2.2*)	
Neutral lean	−10.3 (9.0)	−14.6 (6.9)	**30.4** (**12.2**)	23.4 (25.6)	29.5 (22.8)	**18.7** (**5.7**)
SL onset	*60.9* (*6.7*)	*57.9* (*6.8*)		*56.6* (*3.6*)	*59.0* (*8.3*)	
SL end	*84.3* (*11.6*)	*83.7* (*15.1*)		*86.4* (*3.5*)	*92.8* (*5.5*)	
Posterior lean	62.7 (39.9)	93.4 (57.0)	**26.4** (**2.2**)	63.6 (40.8)	101.7 (80.0)	**53.2** (**19.2**)
SL onset	*68.3* (*5.3*)	*66.1* (*5.7*)		*59.5* (*4.0*)	*57.1* (*3.9*)	
SL end	*93.0* (*3.4*)	*92.9* (*3.6*)		*91.2* (*10.9*)	*93.2* (*9.2*)	

Amplitudes are expressed as a percentage change compared to prestimulus EMG levels. Latencies (in italics) reflect the onset and end of the SL phase. Background activation levels (in bold) are the average of the left and right sides and reflect mean levels across stimuli. Values are expressed as mean (SD)

With sternal stimulation, the polarity of the phases in soleus was inverted for neutral stance and anterior lean with an initial SL inhibition followed by an ML excitation (Fig. 5b). Posterior lean was associated with the largest postural response in tibialis anterior, and a biphasic volley on the unrectified trace was presented in 7/8 subjects (mean peak-to-peak amplitude of 232 μV). For sternal stimulation using the higher (+6 dB) intensity, there was a significant interaction between muscle group and posture for the amplitude (*P* = 0.045) and onset of the SL response (*P* ≪ 0.001) with the onset of the SL response being significantly later for soleus than tibialis anterior in the posterior lean posture (66.1 and 57.1 ms, *P* ≪ 0.001). Posterior lean with sternal stimulation was the only condition for which there was evidence of co-contraction of the tibialis anterior and soleus muscles (Table 4).

**Fig. 6:**
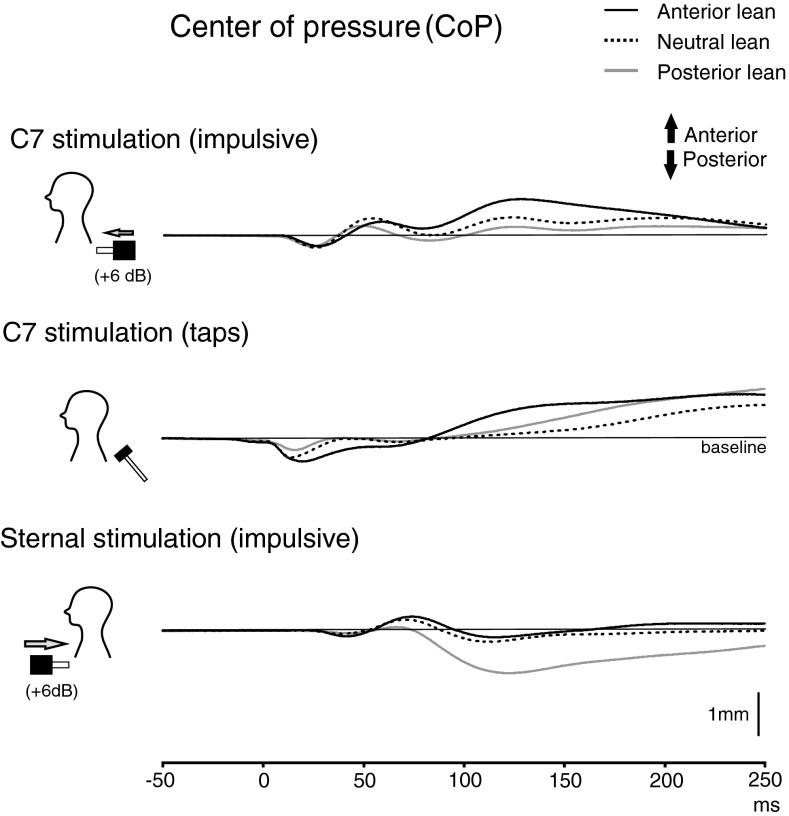
Centre of pressure (CoP) traces for C7 (impulses and taps) and sternal (impulses) stimulation during anterior, neutral and posterior leaning posture (*n* = 8). For impulsive stimuli, anterior lean showed the greatest CoP displacement for C7 stimulation whereas for sternal stimulation the largest was for posterior lean. Taps at C7 evoked CoP displacements that did not return to the prestimulus baseline location during the recording period. The CoP response following impulsive stimuli, in contrast, did return to close to the baseline location by the end of the recordings

Averaging the force platform traces indicated that the peak CoP displacement during C7 stimulation was greatest for anterior lean and progressively decreased in the neutral and posterior leaning postures (anterior: 0.7 ± 0.5 mm, neutral: 0.4 ± 0.3 mm, posterior: 0.2 ± 0.3 mm; *P* = 0.012). Peak CoP displacement during sternal stimulation showed the opposite pattern and was largest during posterior lean (anterior: −0.2 ± 0.2 mm, neutral: −0.3 ± 0.1 mm, posterior: −0.7 ± 0.7 mm; *P* = 0.036). Mean latencies for peak CoP displacement ranged between 117–129 ms for C7 stimulation and 115–118 ms for sternal stimulation. A shorter latency posteroanterior transient showed little change with differing directions of stimulation, and we propose this may represent a transmitted wave evoked by the stimuli. By the end of the recording period, the CoP had returned substantially to the initial position for the impulsive stimuli, but not for the taps (Fig. 6).

Mean near-maximal contraction levels were 84.3 ± 30.4 μV for soleus and 127.0 ± 29.1 μV for tibialis anterior, indicating that anterior and posterior lean led to tonic contractions around 70 % (soleus) and 40 % (tibialis), respectively, of our estimated maximum levels.

## Discussion

We have confirmed the main findings of Graus et al. (2013) and Bőtzel et al. (2001) and have demonstrated that positive (anterior) accelerations of the trunk using impulsive or tendon hammer stimuli evoke SL excitation–inhibition potentials in the soleus muscles. We have confirmed that cutaneous afferents have little or no role in the response and that the responses are unlikely to be of vestibular origin but have also extended these earlier observations. Both vision and surface properties showed little effect on the reflex responses, unlike vestibular-spinal postural reflexes (Welgampola and Colebatch 2001) but this in turn may be partly due to the properties of the perturbations that we used. Horak et al. (1990) showed that somatosensory loss was associated with greater early EMG activity in a variety of leg muscles but that this was only evident when using large displacements. Many reflexes can be expected to scale with background activation (Matthews 1986), but we were able to show the alterations that occurred with specific postural conditions exceeded any changes in tonic activation and thus can be taken to indicate a change of reflex gain.

Head accelerations in the same direction as the trunk, as is normally the case (Graus et al. 2013), produced larger reflex amplitudes than head and trunk accelerations in opposing directions but the type of response was determined by the direction of truncal, rather than head, acceleration. Horak et al. (1994) showed that head displacements alone were sufficient to trigger ML (48–84 ms) responses in leg and trunk muscles. Likewise, we have shown that impulsive lateral accelerations of the head at the mastoids will trigger SL responses in the soleus muscles with properties expected for vestibular reflexes (Laube et al. 2012). In the present series of experiments, we have deliberately dissociated the directions of trunk and head acceleration, with vestibular afferent responses being head-referenced. In all cases, the nature of the postural response in soleus was appropriate for compensating for the direction of truncal acceleration. Horak et al. (1994) also felt that vestibular signals and those from cervical muscles were unlikely to be critical for their postural responses as they too observed dissociations in the direction of head and trunk accelerations as well as smaller responses to head-only displacements. In our study, the average percentage difference between head accelerations concordant and discordant for the direction of truncal acceleration was 11.2 % from which one can estimate an average effect of vestibular activation of half this amount (5.6 %), which is less than a quarter of the usual degree of excitation related to truncal stimulation (27 % on average for the 0 dB stimulus—Table 3). The SL responses reported by Laube et al. (2012) using a mastoid stimulus to excite vestibular reflexes were around 8.0 % of the background activation, consistent with the weaker effect of vestibular reflexes. Horak et al. (1994) reported that responses to head displacements were about one-third the size of those to truncal perturbations. We conclude that the responses that we have shown do not primarily originate in vestibular receptors but when both the head and the trunk accelerate in the same direction, their evoked postural responses reinforce.

The reflex responses were not unique to a particular muscle group, given that similar responses could also be elicited from the hamstrings. Both responses act to stabilise the trunk in response to the applied disturbance which tended to flex the trunk, by extending the ankles and the hips. While it is often stated that greatest sway when standing normally occurs around the ankle joints, direct measurements have not supported this (Day et al. 1993). Our findings indicate, however, that the most distant joint from the centre of body mass is the main focus for control of posture for the anteroposterior plane both when standing (calf muscles) and kneeling (thigh muscles). The size of the reflex responses was substantially larger for soleus than for the hamstrings for normal stance. Hamstring responses were much larger during kneeling than standing, consistent with their greater role in postural stability under these conditions. Kneeling attenuated the responses in the soleus but did not abolish them even though the soleus muscles now had no role in stabilising the trunk. During kneeling, posture should not influence proprioceptive input from the ankle joint, indicating that the reflexes evoked by postural disturbances under these conditions do not originate from ankle proprioceptors. The changes with kneeling are likely to represent a change in gain of the reflex as they were not the result of changes in tonic activation. Notable also were the repeated bursts of activity which were present in the hamstrings in response to some perturbations, which would have increased their effectiveness in stabilising the trunk. These might contribute to the greater high frequency oscillations reported for kneeling compared to normal stance (Mezzarane and Kohn 2008).

Leaning forwards and backwards to deliberately destabilise our subjects also had profound effects on the gain and nature of the responses. In general, EMG responses were enhanced for only the stimulus conditions that posed the greatest threat to postural stability and for the muscle group most important for compensating for the truncal displacement. Similar findings were previously reported by Bőtzel et al. (2001), using sternal taps. For soleus this was during anterior lean and for C7 stimulation, whereas for tibialis anterior the greatest enhancement was during posterior lean and sternal stimulation. In both cases, the muscle responses were enhanced specifically for the perturbation tending to cause sway in the direction of least stability. We also found evidence for a functional role for the reflex responses to disturbances during postural lean, given that the CoP traces returned to near the initial position, but does this truncal reflex have a role in normal standing, when spontaneous accelerations are smaller? We have no definitive evidence about this, but there is some indirect support for such a role. Day et al. (1993) found that the largest angular motion in normal standing was usually between the trunk and the leg, indicating that truncal reflexes could be evoked during normal stance and contribute to its stabilisation. Such a role would explain the otherwise surprising observation by Fitzpatrick et al. (1994) that sway increased when body segments were splinted, reducing movement at joints other than the ankles (see also Mok and Hodges 2013).

The responses that we recorded were clearly reflexes based upon the latency and the fact that our subjects were not aware of the direction of stimulation and therefore could not have voluntarily reacted to it. We are not the first to propose a postural reflex evoked by truncal accelerations. Previous authors have found evidence for postural reflexes evoked by truncal displacements and have speculated as to their possible origin. Do et al. (1988) for example, studied subjects leaning forwards who were suddenly released. They showed reflex responses beginning in soleus at an average of 59 ms after release, similar to our latencies. Vestibular disorders and ischaemia of the leg did not affect the responses, and they suggested a role for the intrinsic back muscles. Gurfinkel et al. (1981) showed postural responses in soleus were related to truncal orientation, not the degree of shortening of the soleus muscles. They also showed that the responses persisted despite immobilisation of the ankles or head. The receptors responsible for the reflex responses must be very sensitive to small displacements and also able to signal the direction of imposed acceleration. The extensor truncal muscles are richly innervated by muscle spindles (Kokkorogiannis 2004), and we also propose that these may be responsible. The reduced effectiveness of a short tap compared to a slightly more prolonged stimulus is consistent with the co-activation of muscle spindles that may occur with brief tap stimuli (e.g. Colebatch et al. 2014). Given the latency of the responses, similar to that for vestibular-spinal postural reflexes, a spinal-bulbar-spinal pathway seems most likely. It also seems plausible that the efferent limb is mediated via the reticulospinal tract, similar to proposals for vestibular-spinal reflexes (Britton et al. 1993). The medial reticulospinal tract is rapidly conducting and makes monosynaptic connections with neck, axial and limb motoneurones, and individual projection fibres make widespread contacts (Wilson and Peterson 1981). The latter property may explain why soleus activity was still present when kneeling. Reticulospinal activity may also occur in repeated bursts, perhaps analogous to the repeated responses seen in the hamstrings when kneeling (Weinberger et al. 2008).

We have provided evidence that the postural reflex evoked by brief axial accelerations does not arise from vestibular receptors or from proprioceptors at the ankle. It does not depend upon cutaneous receptors and shows little change in response to vision or surface characteristics. It is however strongly modulated by both postural task and truncal orientation, being greatly enhanced when there is a direct threat to postural stability.

## Electronic supplementary material

Below is the link to the electronic supplementary material.

Supplementary Figure—grand mean EMG recordings from the soleus muscle group before (grey) and after (black) application of local anaesthesia (n = 8) to the site of stimulation. Standard (0 dB) and high (+6 dB) intensity impulsive stimuli and taps were used to elicit responses in the standing and kneeling positions. There was no significant effect of local anaesthesia on the latency or amplitude of the responses in either the soleus or hamstrings muscles (PDF 1342 kb)

Supplementary material 2 (DOC 45 kb)
