# Supplementary material for: Axially evoked postural reflexes: influence of task
Source: Exp Brain Res. 2014 Oct 10;233(1):215–28. doi: 10.1007/s00221-014-4105-8 (PMC4289977; doi:10.1007/s00221-014-4105-8)
Supplement: Supplementary file 1 — Supplementary Figure—grand mean EMG recordings from the soleus muscle group before (grey) and after (black) application of local anaesthesia (n = 8) to the site of stimulation. Standard (0 dB) and high (+6 dB) intensity impulsive stimuli and taps were used to elicit responses in the standing and kneeling positions. There was no significant effect of local anaesthesia on the latency or amplitude of the responses in either the soleus or hamstrings muscles (PDF 1342 kb) [file 221_2014_4105_MOESM1_ESM.pdf]

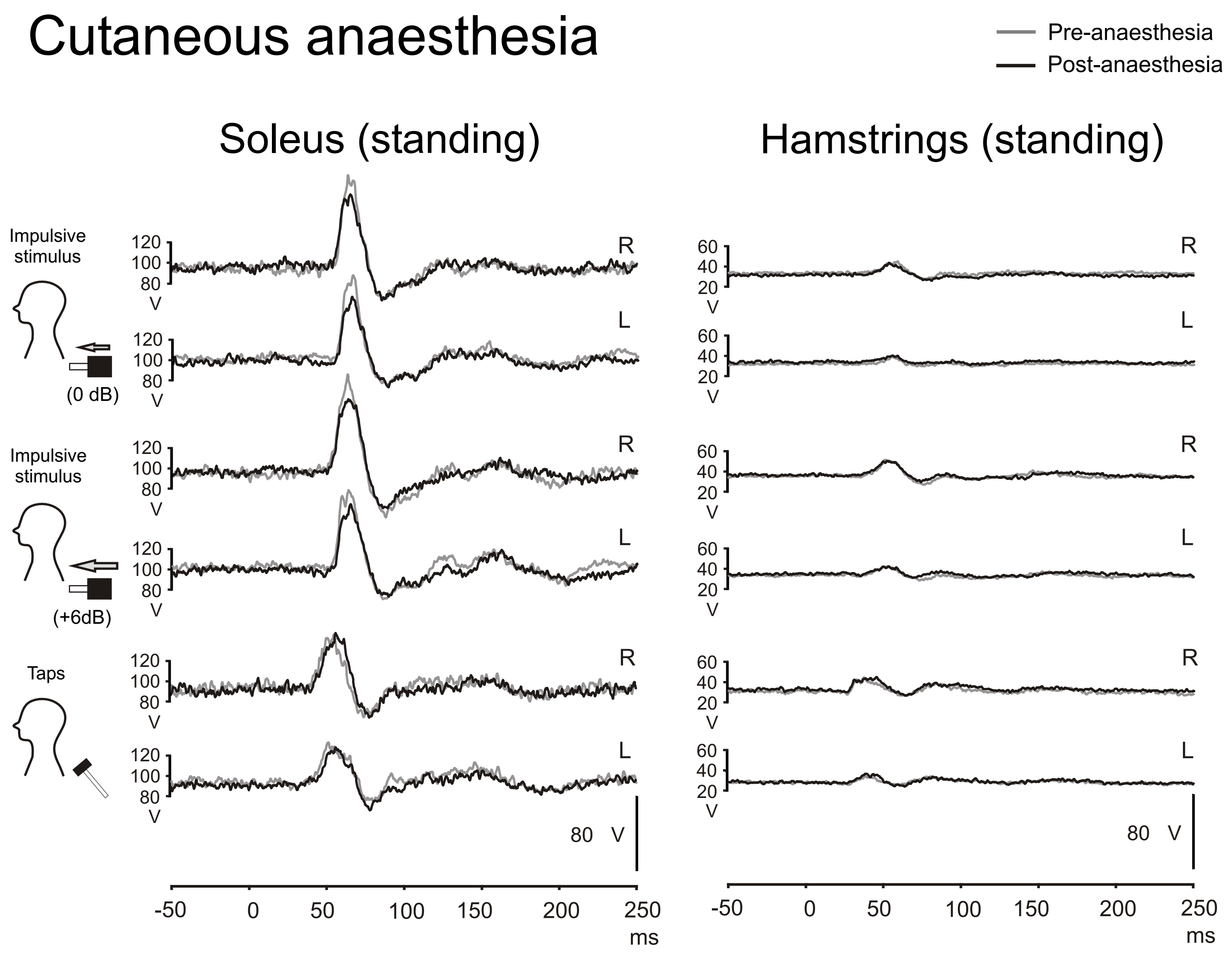

Cutaneous anaesthesia
Pre-anaesthesia
Post-anaesthesia
Soleus (standing)
Hamstrings (standing)
Impulsive stimulus
(0 dB)
Impulsive stimulus
(+6dB)
Taps
R
L
120
100
80
V
60
40
20
80 V
-50
0
50
100
150
200
250
ms
