# Supplementary material for: Axially evoked postural reflexes: influence of task
Source: Exp Brain Res. 2014 Oct 10;233(1):215–28. doi: 10.1007/s00221-014-4105-8 (PMC4289977; doi:10.1007/s00221-014-4105-8)
Supplement: Supplementary file 2 — Supplementary material 2 (DOC 45 kb) [file 221_2014_4105_MOESM2_ESM.doc]

**Supplementary Table: Acceleration amplitudes and peak latencies in the standing and kneeling conditions**

|  | ***Head*** | | | |  | ***Trunk*** | | | |
| --- | --- | --- | --- | --- | --- | --- | --- | --- | --- |
|  | *Impulsive*  *stimulus*  *(0dB)* | *Impulsive*  *stimulus*  *(+6dB)* | | *Taps* |  | *Impulsive*  *stimulus*  *(0dB)* | *Impulsive*  *stimulus*  *(+6dB)* | | *Taps* |
|  | EC | EC | EO | EC |  | EC | EC | EO | EC |
| ***Standing*** |  |  |  |  |  |  |  |  |  |
| ***Rigid surface*** |  |  |  |  |  |  |  |  |  |
| Amplitude  (m*g*) | 23.5  (9.7) | 44.1  (22.9) | 44.0  (23.1) | 80.2  (61.4) |  | 38.3  (19.8) | 86.5  (44.9) | 89.2  (50.1) | 354.0  (242) |
| Latency  (ms) | 14.8  (3.6) | 14.5  (4.0) | 15.7  (4.2) | 9.3  (3.3) |  | 10.8  (1.9) | 11.2  (1.8) | 11.9  (1.9) | 8.1  (1.0) |
| ***Compliant surface*** |  |  |  |  |  |  |  |  |  |
| Amplitude  (m*g*) | 22.6  (11.0) | 45.0  (24.1) | - | 70.3  (69.8) |  | 43.0  (24.7) | 92.4  (48.6) | - | 382.2  (243.8) |
| Latency  (ms) | 15.6  (3.6) | 15.1  (4.2) | - | 8.6  (2.8) |  | 11.1  (1.7) | 11.6  (1.9) | - | 8.3  (0.8) |
| ***Kneeling*** |  |  |  |  |  |  |  |  |  |
| Amplitude  (m*g*) | 27.6  (7.8) | 52.5  (18.7) | - | 80.0  (62.9) |  | 43.8  (20.3) | 99.5  (48.1) | - | 402.5  (262.5) |
| Latency  (ms) | 14.9  (3.0) | 14.9  (3.1) | - | 11.0  (6.4) |  | 10.0  (3.4) | 11.0  (2.6) | - | 9.0  (13.4) |

**EC = eyes closed, EO = eyes open, Values are expressed as mean (SD)**
